# Identification of Potential Biomarkers and Immune Infiltration Characteristics in Idiopathic Pulmonary Arterial Hypertension Using Bioinformatics Analysis

**DOI:** 10.3389/fcvm.2021.624714

**Published:** 2021-02-01

**Authors:** Haowei Zeng, Xiaoqin Liu, Yushun Zhang

**Affiliations:** Department of Structural Heart Disease, The First Affiliated Hospital of Xi'an Jiaotong University, Xi'an, China

**Keywords:** idiopathic pulmonary arterial hypertension, immune infiltration, inflammation, biomarkers, bioinformatics

## Abstract

**Objectives:** Idiopathic pulmonary arterial hypertension (IPAH) is a rare but severe lung disorder, which may lead to heart failure and early mortality. However, little is known about the etiology of IPAH. Thus, the present study aimed to establish the differentially expressed genes (DEGs) between IPAH and normal tissues, which may serve as potential prognostic markers in IPAH. Furthermore, we utilized a versatile computational method, CIBERSORT to identify immune cell infiltration characteristics in IPAH.

**Materials and Methods:** The GSE117261 and GSE48149 datasets were obtained from the Gene Expression Omnibus database. The GSE117261 dataset was adopted to screen DEGs between IPAH and the control groups with the criterion of |log2 fold change| ≥ 1, adjusted *P* < 0.05, and to further explore their potential biological functions via Gene Ontology analysis, Kyoto Encyclopedia of Genes and Genomes Pathway analysis, and Gene Set Enrichment Analysis. Moreover, the support vector machine (SVM)-recursive feature elimination and the least absolute shrinkage and selection operator regression model were performed jointly to identify the best potential biomarkers. Then we built a regression model based on these selected variables. The GSE48149 dataset was used as a validation cohort to appraise the diagnostic efficacy of the SVM classifier by receiver operating characteristic (ROC) analysis. Finally, immune infiltration was explored by CIBERSORT in IPAH. We further analyzed the correlation between potential biomarkers and immune cells.

**Results:** In total, 75 DEGs were identified; 40 were downregulated, and 35 genes were upregulated. Functional enrichment analysis found a significantly enrichment in heme binding, inflammation, chemokines, cytokine activity, and abnormal glycometabolism. *HBB, RNASE2, S100A9*, and *IL1R2* were identified as the best potential biomarkers with an area under the ROC curve (AUC) of 1 (95%CI = 0.937–1.000, specificity = 100%, sensitivity = 100%) in the discovery cohort and 1(95%CI = 0.805–1.000, specificity = 100%, sensitivity = 100%) in the validation cohort. Moreover, immune infiltration analysis by CIBERSORT showed a higher level of CD8+ T cells, resting memory CD4+ T cells, gamma delta T cells, M1 macrophages, resting mast cells, as well as a lower level of naïve CD4+ T cells, monocytes, M0 macrophages, activated mast cells, and neutrophils in IPAH compared with the control group. In addition, *HBB, RNASE2, S100A9*, and *IL1R2* were correlated with immune cells.

**Conclusion:** *HBB, RNASE2, S100A9*, and *IL1R2* were identified as potential biomarkers to discriminate IPAH from the control. There was an obvious difference in immune infiltration between patient with IPAH and normal groups.

## Introduction

Pulmonary arterial hypertension (PAH) is a progressive cardiopulmonary disease in which increased pulmonary arterial pressure leads to right ventricle failure and premature death. PAH is defined as mean pulmonary artery pressure (mPAH) ≥ 25 mmHg, pulmonary artery wedge pressure ≤ 15 mmHg and pulmonary vascular resistance (PVR)> 3 Wood units. There are several categories of reasons for patients to develop PAH, but in the absence of any known triggering factor, they are labeled as idiopathic PAH (IPAH) (1). It is progressive and affects the precapillary pulmonary vasculature. It is rare but fatal with a high mortality rate, and its prevalence accounts vary among the medical literature (2). IPAH may lead to increased PVR, progressive right ventricular dysfunction, right heart failure, and early mortality. The symptoms of IPAH are non-specific, including fatigue, weakness, shortness of breath, angina, and syncope, usually induced by exertion (1, 3, 4). Although the cause of IPAH is unclear, some cases are familial and caused by gene defects, or mutations, such as *BMPR2* (5), *SMAD8* (6), *ALK1* (7), and *BMPR1B* (8) and so on. Additionally, environmental factors, such as acute hypoxia may be involved in IPAH (9). The diagnosis of IPAH requires the identification of symptoms and a physical examination and a comprehensive set of investigations to guarantee that hemodynamic criteria are met and to describe the etiology as well as the functional and hemodynamic severity of the condition. Despite extensive researches and improved diagnosis as well as a broad range of increasing biomarkers, there is currently no specific marker for IPAH. This disorder is still associated with a poor prognosis, especially when complicated with arrhythmia, left ventricular dysfunction, or heart failure. Therefore, prompt and specific diagnoses are needed to reduce mortality rates.

Although the specific pathophysiology remains poorly understood, increasing evidence suggests a crucial role of inflammation in IPAH. Patients with IPAH showed an accumulation of immune cells around the vessel, including dendritic cells (DCs), B and T lymphocytes, macrophages, and mast cells. Additionally, circulating levels of several chemokines and cytokines are also increased (10–15).

Recently, gene expression microarray studies have been extensively applied to find potential biomarkers and their roles in complicated diseases to further explore the pathogenesis and develop potential treatments (16). In this study, the GSE117261 dataset was adopted to screen differentially expressed genes (DEGs) between IPAH and a control group, and to further explore their potential biological functions via enrichment analysis. We identified the best feature genes that could distinguish IPAH from controls. Finally, immune infiltration was investigated in IPAH by CIBERSORT (17).

## Materials and Methods

### IPAH Datasets

The GSE117261 and GSE48149 datasets were downloaded from the NCBI Gene Expression Omnibus (GEO) database (https://www.ncbi.nlm.nih.gov/geo/). GSE117261 was based on the GPL6224 Affymetrix Human Gene 1.0 ST Array. It consists of transcript profiles of lung tissues from 25 normal individuals and 32 patients with IPAH, which served as a discovery cohort. Its expression value was normalized using the “*limma*” package (18) in R software (version 4.0.2; https://www.r-project.org/) to make sure the expression levels had a similar distribution among a set of arrays. GSE48149 was based on GPL16221 Illumina HumanRe-8 v3.0 expression beadchip. The lung tissues of nine normal individuals and eight patients with IPAH were obtained during lung surgery, which served as a validation cohort. Its expression level was normalized and log2 transformed by R software.

### Identification of DEGs

To establish the DEGs between the IPAH and the control group, the “*limma*” package (18) was adopted in R software. Moreover, a volcano plot was generated to assess the DEGs, |log2 fold change (FC) |≥ 1, adjusted *P* value < 0.05 was set as a cutoff for this selection.

### Functional Enrichment of DEGs

The Gene Ontology (GO) analysis, Kyoto Encyclopedia of Genes and Genomes (KEGG) pathway analysis, and Gene Set Enrichment Analysis (GSEA) were carried out to annotate and analyze the biological functions of the DEGs. The GO project provides a set of hierarchical controlled vocabulary divided into three categories: cellular component, biological process and molecular function. The KEGG pathway is a group of pathway maps showing the knowledge of the molecular interaction, reaction and relation networks. GSEA is a computational approach that decides whether an a *priori* defined set of genes shows statistically significant differences between two groups. R software and the “*clusterProfiler*” package (19) were used to conduct GO analysis and KEGG analysis. In this analysis symbol codes were converted to Entrez ID using Human genome annotation package “*org.Hs.eg.db*.” We used the “*ggplot2*” (20), “*pathview*” (21) and “*gplots”* packages of R software to visualize the plots. GSEA software (version 4.1.0, http://www.gsea-msigdb.org/gsea/index.jsp) was used to carry out GSEA analysis (22). The “c2.cp.kegg.v7.0.symbols.gmt” was chosen as the reference gene set. Gene set permutations were performed 1,000 times for each analysis. Adjusted *P-*value < 0.05 and *Q* value < 0.05 were considered significant for GO and KEGG analysis. The cutoff point of significance was |normalized enrichment score (NES)|>1, *P-*value < 0.05, false discovery rate (FDR) *Q* value < 0.25 for GSEA.

### Feature Gene Identification and Support Vector Machine Classifier Construction

Support vector machine (SVM) is a supervised machine learning algorithm that can be used for regression or classification, which is derived on the basis of a training cohort that detects patterns in the data and associates them with labels (23). The SVM -recursive feature elimination (RFE) is a machine learning algorithm requires training multiple classifiers on subsets of features of decreasing size to search for the best variable (24). The least absolute shrinkage and selection operator (LASSO) regression model was further conducted to select optimal variables with the use of penalty coefficient. The LASSO logistic regression method and the SVM-RFE were used to select the most significant feature genes and build a regression model using the selected variables (25, 26). The performance of the SVM classifier in distinguishing IPAH from control groups was assessed by the area under the receiver operating characteristic (ROC) curve (AUC) in the discovery and validation cohorts. The “*glmnet*” package (27) was adopted to analyze LASSO regression. The “*e1071*” package (28) was used to perform the SVM algorithm. The “*pheatmap*” package (29) was used to construct a heat map of genes for all samples in both the discovery and validation cohorts.

### Immune Infiltration Analysis

CIBERSORT, a method of analyzing the different immune cell types of tissues using their gene expression profiles (30), was adopted to analyze the normalized GSE117261 expression data. Next, a matrix of 22 kinds of immune cells was obtained. CIBERSORT *P* < 0.05 was used to filter the samples. And the percentage of each immune cell type in the samples was calculated and displayed in a bar plot. A heat map of the 22 immune cells was constructed using the “*pheatmap*” package (29). The “*vioplot*” package (31) was used to compare the levels of 22 immune cells between the two groups. A correlation heatmap, which revealed the correlation of 22 kinds of immune cells, was made by the “*corrplot*” package (32) of R software. The “*ggplot2*” package (20) was adopted to visualize the plots.

### Correlation Analysis Between Immune Cells and Feature Genes

Spearman's rank correlation analysis was used to analyze the relationship between immune cells and feature genes in R software. The “ggplot2” package (20) was adopted to visualize the plot, *P-*value < 0.05 was considered statistically significant.

### Statistical Analysis

R software (version 4.0.2; https://www.r-project.org/) was used for all statistical analysis. Continuous variables are expressed as mean ± SD, and differences between two groups were compared using Student's *t*-test for normally distributed variables and Mann–Whitney U test for abnormally distributed variables. The sensitivity and specificity of feature genes to distinguish IPAH from controls were assessed using a ROC curve. Adjusted *P-*value < 0.05 was considered statistically significant.

## Results

### Data Preprocessing and Identification of DEGs

**Figure 1:**
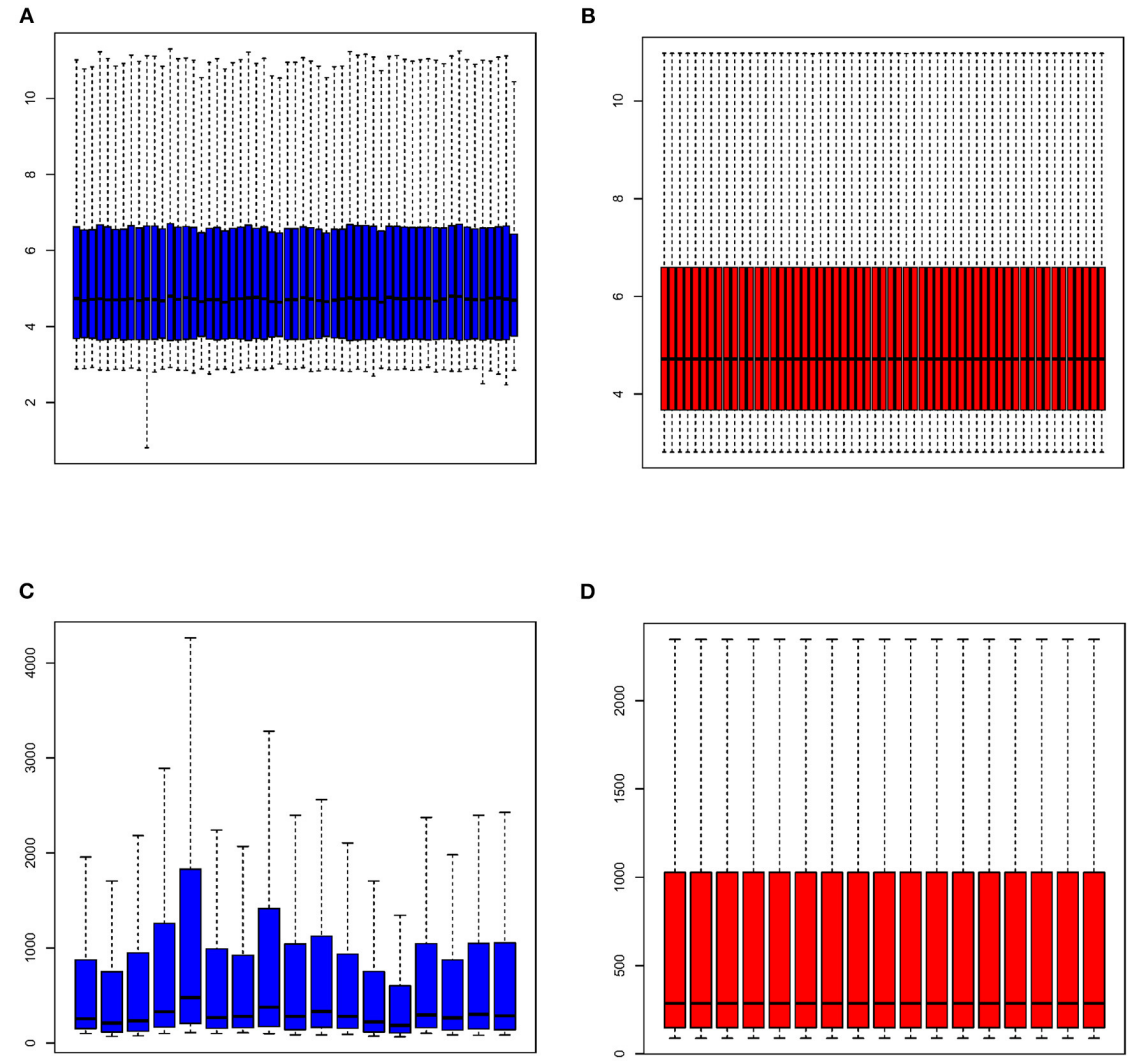
The box diagram of expression profile before and after normalization. GSE117261 expression profile before **(A)** and after **(B)** normalization; GSE48149 expression profile before **(C)** and after **(D)** normalization.

**Figure 2:**
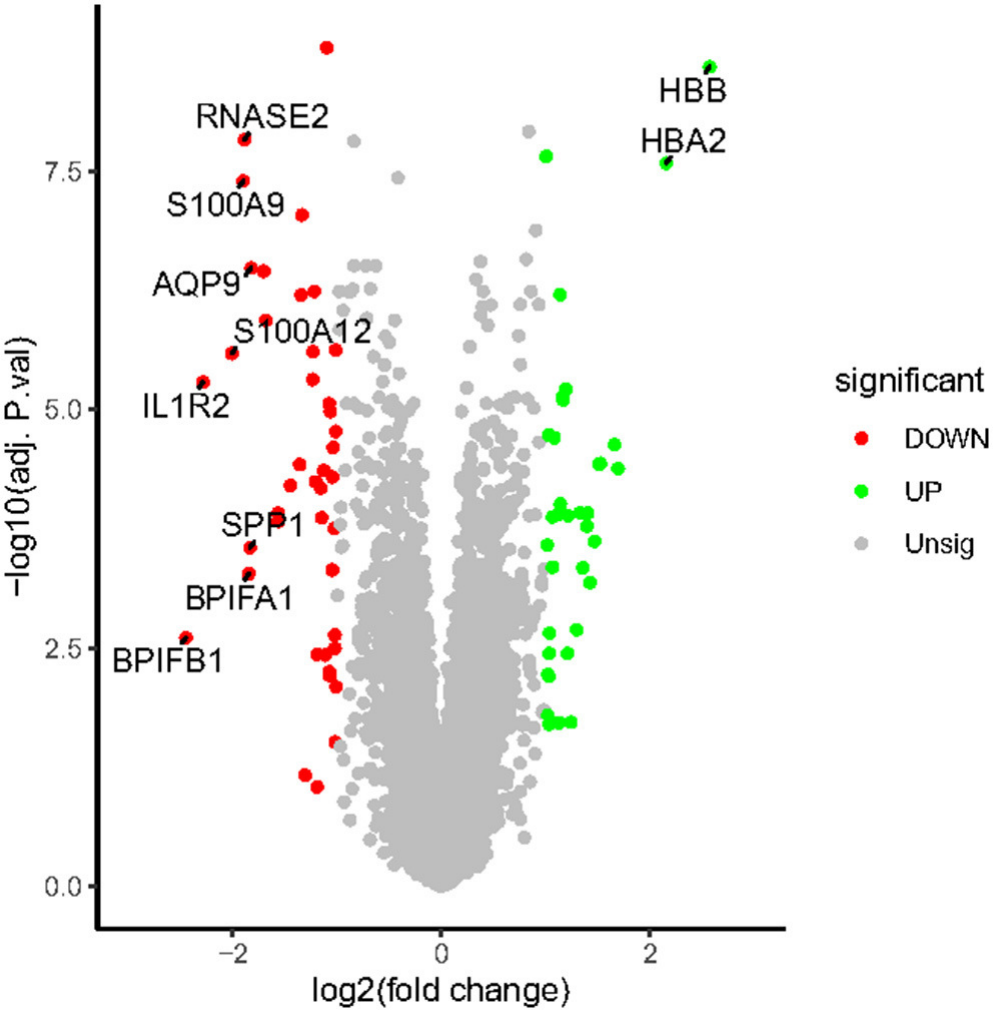
The volcano map of DEGs. The plot compared the DEGs between IPAH patients and controls from the GSE117261 dataset. The red dots represent the down-regulated DEGs; the green dots represent up-regulated DEGs; gray dots indicate the remaining genes that were not significantly changed. Genes signed in the plot are the top ten genes with the most significant logFC.

All expression values of the GSE117261 and GSE48149 datasets were normalized. And the data before and after normalization are presented as box diagrams in Figure 1. In total, 75 DEGs were identified: 40 were downregulated and 35 genes were upregulated. The volcano plot showed (Figure 2) that *BPIFB1, IL1R2, S100A12, S100A9, RNASE2, BPIFA1, SPP1, AQP9, HBA2*, and *HBB* were the top ten genes with the most significant logFC, and were used to further identify potential biomarkers.

### Functional Enrichment Analysis of DEGs

**Figure 3:**
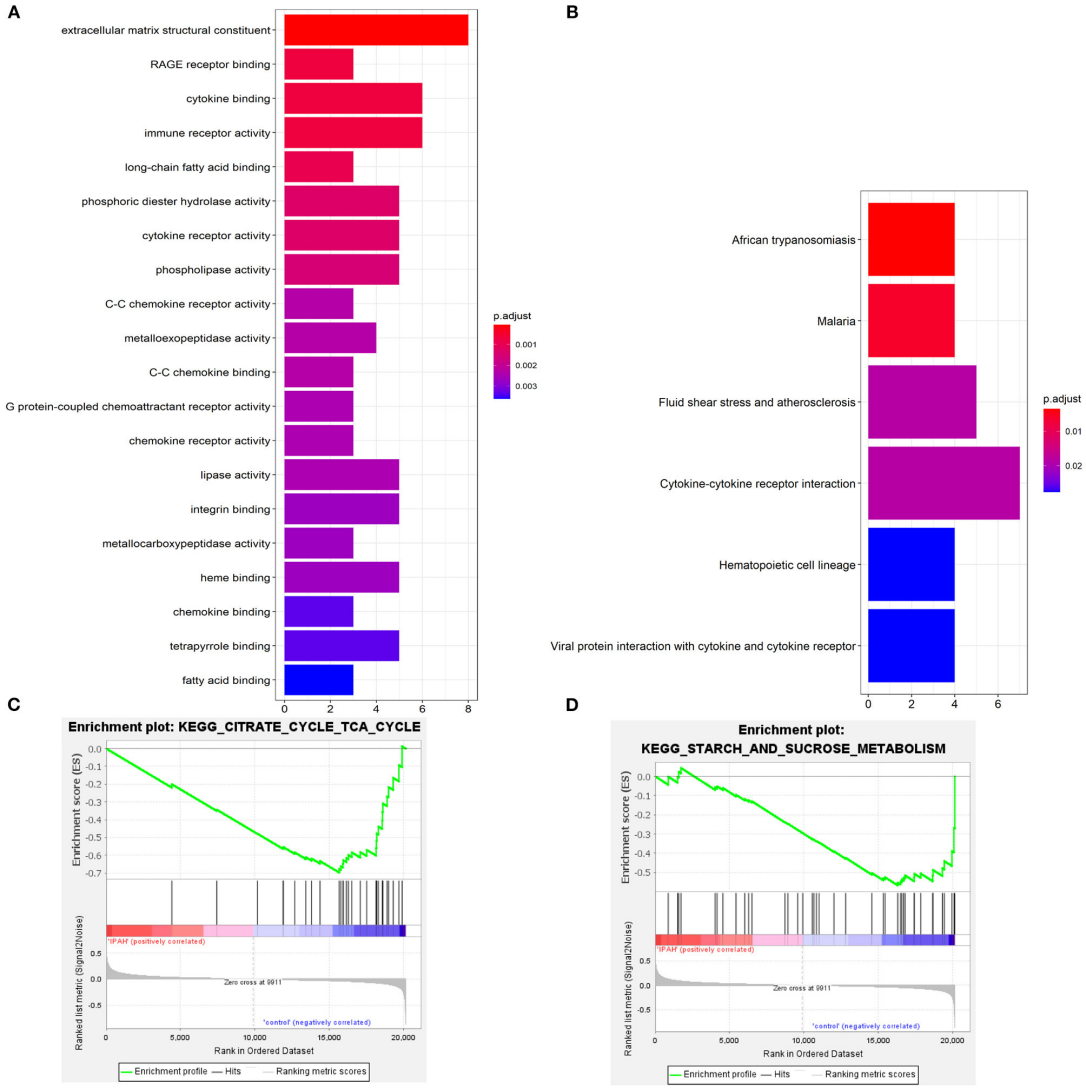
The functional enrichment analysis of DEGs. **(A)** The GO enrichment; **(B)** The KEGG enrichment; **(C,D)** The GSEA enrichmen analysis.

Go enrichment analysis demonstrated that DEGs were mainly associated with heme binding (GO:0020037), chemokine binding (GO:0019956), cytokine binding (GO:0019955), immune receptor activity (GO:0140375), cytokine receptor activity (GO:0004896), C-C chemokine binding (GO:0019957), C-C chemokine receptor activity (GO:0016493), and chemokine receptor activity (GO:0004950) (Figure 3A). The KEGG enrichment analysis was primarily involved in fluid shear stress and atherosclerosis (hsa05418), cytokine-cytokine receptor interaction (hsa04060), viral protein interaction with cytokine and cytokine receptor (hsa04061) (Figure 3B). In the GSEA analysis, the citrate cycle (TCA cycle) (hsa00020, NSE = −1.83, normal *P-*value = 0.004, FDR = 0.108) and starch and sucrose metabolism (hsa00500, NSE = −1.66, normal *P*-value = 0.004, FDR = 0.204) were remarkably suppressed in patients with IPAH (Figures 3C,D). In summary, functional enrichment showed the DEGs mainly involved in heme binding, inflammation, chemokine, cytokine activity, and abnormal glycometabolism.

### Feature Genes Selection and SVM Classifier Construction

**Figure 4:**
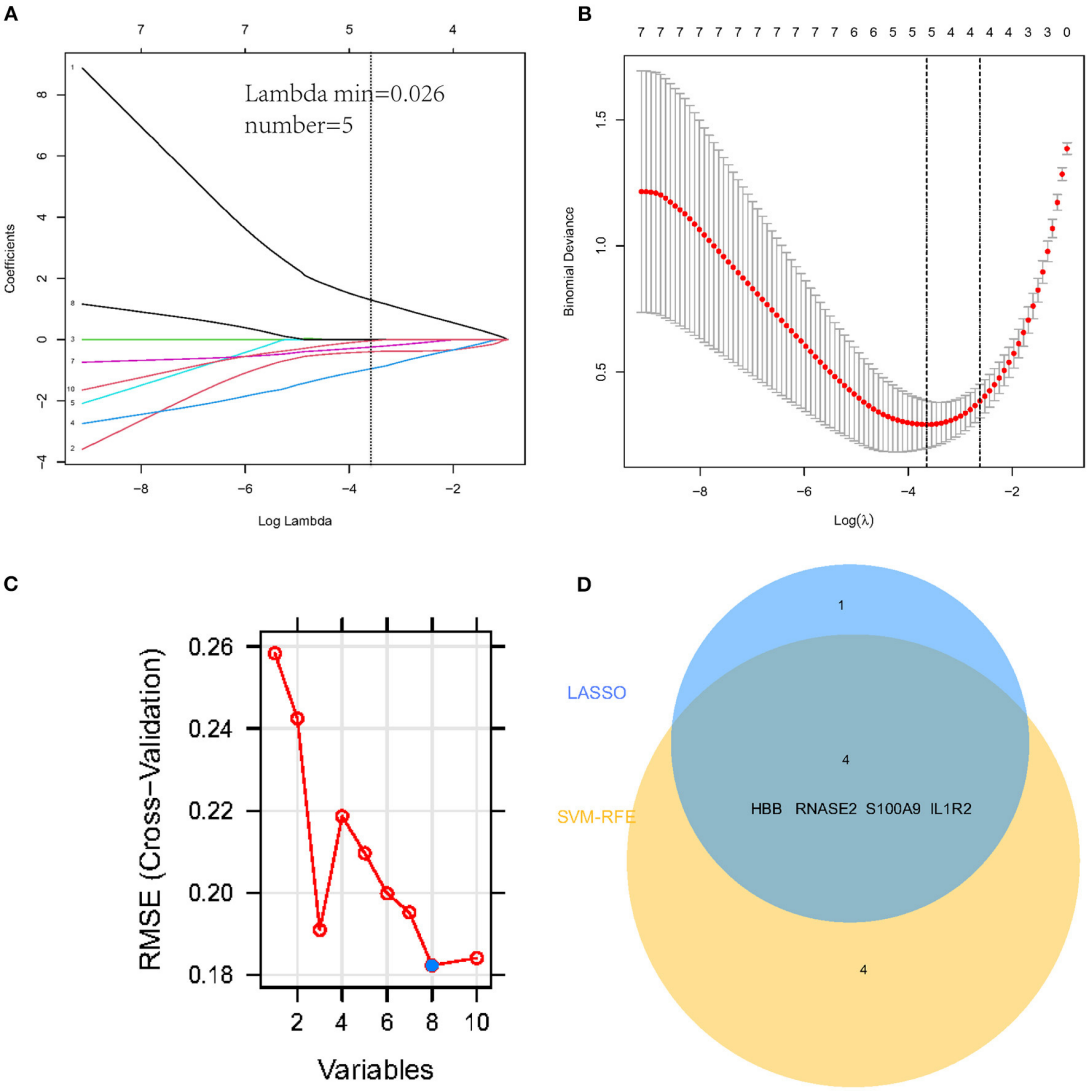
Establishment of prognostic genes signature by LASSO regression analysis and SVM-RFE algorithm. **(A)** LASSO coefficient profiles of five genes. The dotted vertical line is the value selected using the 10-fold cross-validation in **(B)**, for which the optimal lambda λ) resulted in five non-zero coefficients. **(B)** Identification of the optimal penalization coefficient (λ) in the Lasso model used 10-fold cross-validation and the minimum absolute contraction criterion. **(C)** A plot of feature gene selection by SVM-RFE. The blue dot represents the best eight variables; **(D)** The Venn plot of overlapped feature genes between LASSO regression analysis and SVM-RFE algorithm.

**Figure 5:**
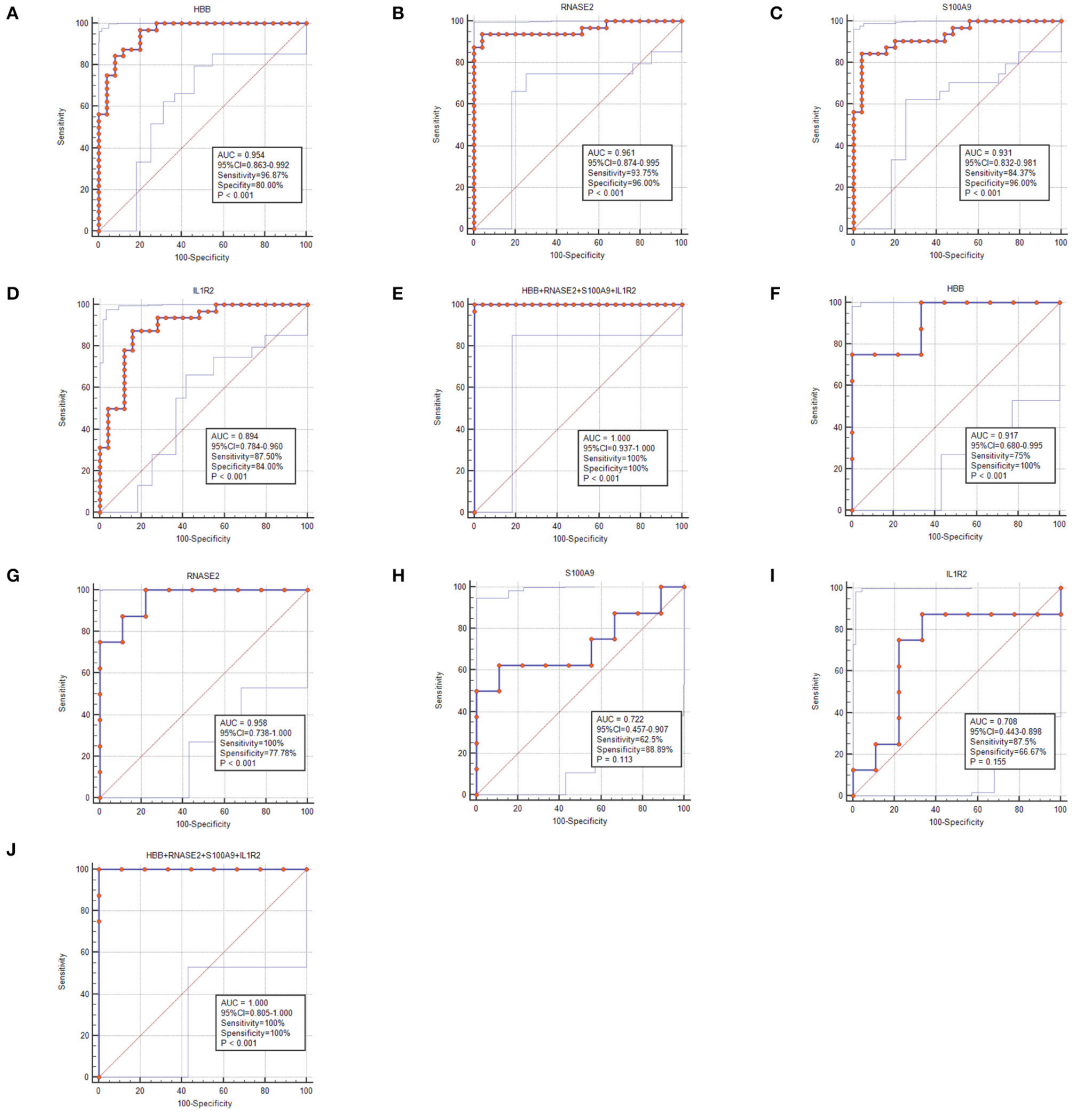
The receiver operating characteristic (ROC) curve of the diagnostic effectiveness of the feature genes. **(A–E)** ROC curve of *HBB, RNASE2, S100A9, IL1R2*, and support vector machine (SVM) classifier in GSE117261; **(F–J)** ROC curve of *HBB, RNASE2, S100A9, IL1R2*, and SVM classifier in GSE48149.

**Figure 6:**
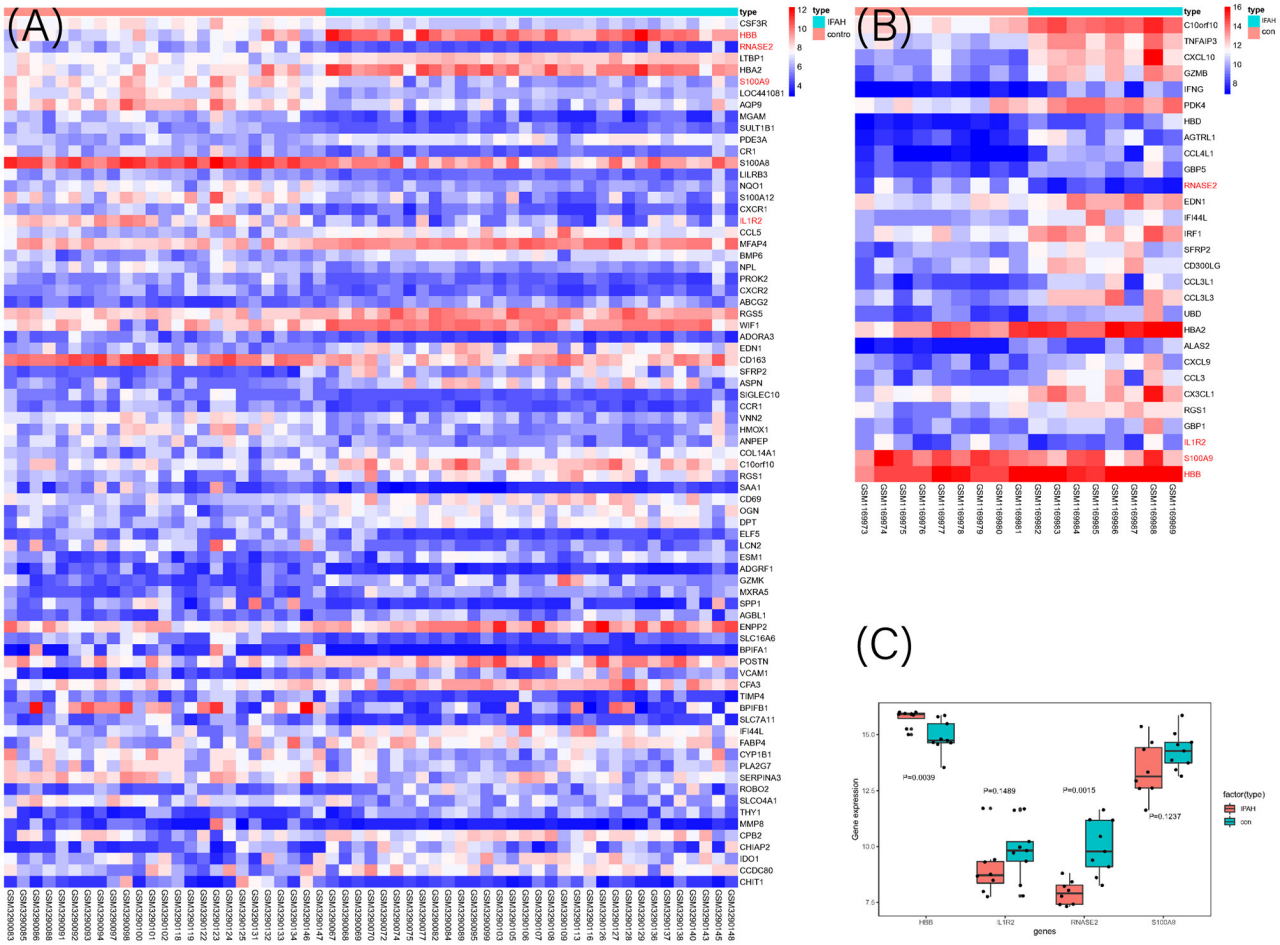
The heat map of DEGs in discovery cohort **(A)** and validation cohort **(B)** between IPAH and the control group. The selected four genes were marked as red color; **(C)** The expression value of the identified four genes in the validation cohort.

To select the best biomarkers of IPAH, the top ten genes with the most significant logFC were further analyzed. The LASSO logistic regression method was used to identify five potential biomarkers (Figures 4A,B): *HBB, RNASE2, S100A9, IL1R2*, and *BPIFB1*. The SVM-RFE identified eight (Figure 4C): *RNASE2, HBB, S100A9, HBA2, SPP1, AQP9, IL1R2*, and *S100A12*. Eventually, we got four diagnostic genes by the two algorithms overlapped (Figure 4D): *HBB, RNASE2, S100A9*, and *IL1R2*, of which *HBB* was upregulated, whereas *RNASE2, S100A9*, and *IL1R2* were downregulated. Then, the ROC analysis was used to further appraise the predictive value of the four feature genes, respectively, and jointly in the diagnosis of IPAH in the two cohorts. The AUC was 0.954 (95%CI = 0.863–0.992) for *HBB*, 0.961 (95%CI = 0.874–0.995) for *RNASE2*, 0.931 (95%CI = 0.832–0.981) for *S100A9*, 0.894 (95%CI = 0.784–0.960) for *IL1R2*, and 1 (95%CI = 0.937–1.000) when these four genes combined into one variable (Figures 5A–E) in GSE117261 dataset. And the AUC was 0.917 (95%CI = 0.680–0.995) for *HBB*, 0.958 (95%CI = 0.738–1.000) for *RNASE2*, 0.722 (95%CI = 0.457–0.907) for *S100A9*, 0.708 (95%CI = 0.443–0.898) for *IL1R2*, and 1 (95%CI = 0.805–1.000) when these four genes combined into one variable in GSE48149 dataset (Figures 5F–J). Hierarchical clustering analysis in both the discovery (Figure 6A) and validation cohorts (Figure 6B) suggested that the patients were clearly divided into two groups based on the expression levels of these four DEGs. Figure 6C showed the different expression level of these four genes between IPAH and the control in the validation cohort. The result suggested that these four genes have a good diagnostic value for discriminating IPAH from the control group.

### Immune Infiltration Analysis

**Figure 7:**
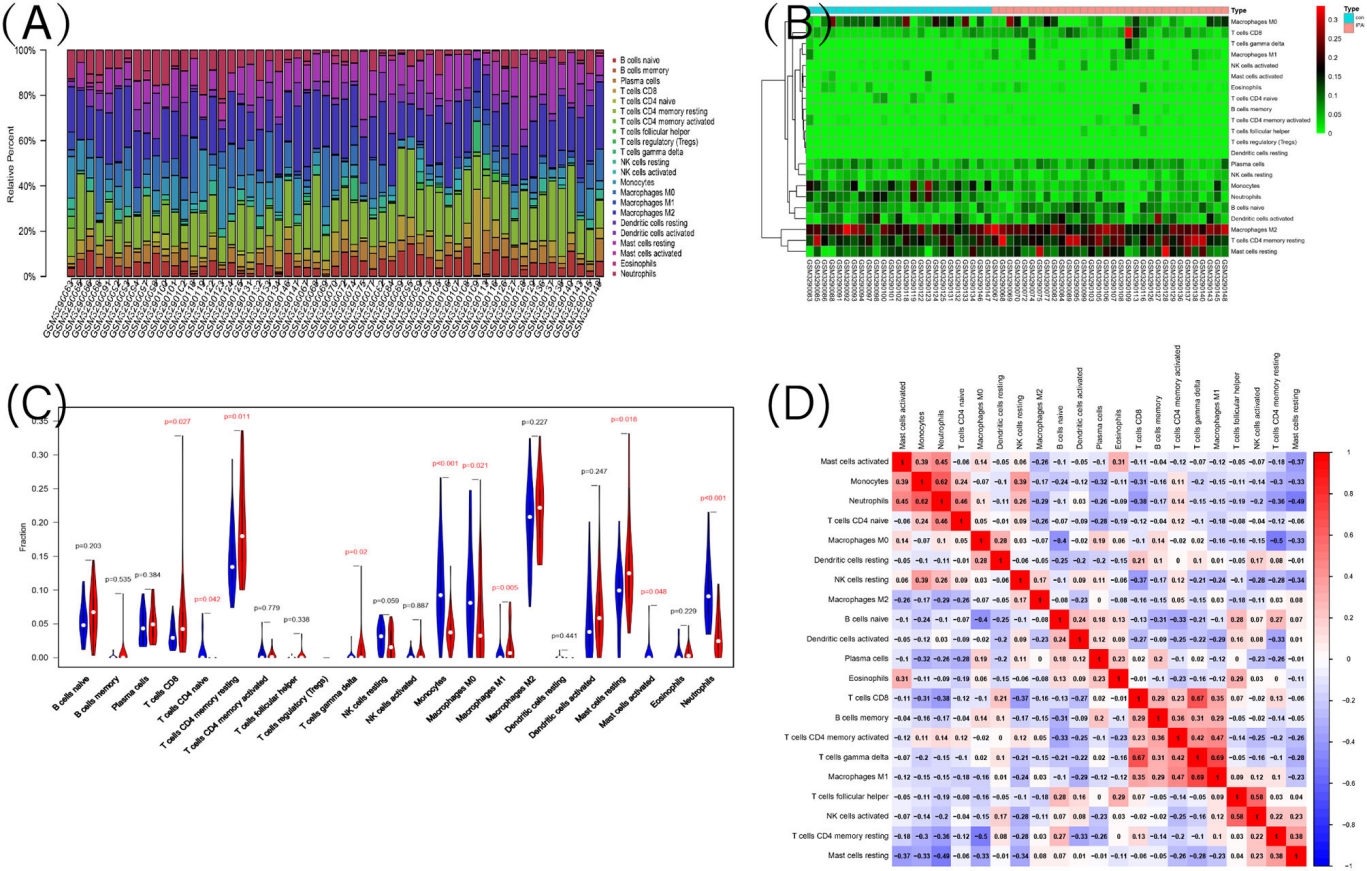
The landscape of immune infiltration between IPAH and normal controls. **(A)** The relative percentage of 22 types of immune cells; **(B)** The heat map of 22 types of immune cells; **(C)** The difference of immune infiltration between IPAH and normal controls. The normal control group was marked as blue color and IPAH group was marked as red color. *P-*values < 0.05 were considered as statistically significant; **(D)** Correlation heatmap of the 22 types of immune cells. Blue presents a negative correlation, red represents a positive correlation, the darker the color, the stronger the correlation.

Functional enrichment analysis of DEGs showed an obvious enrichment in immune response. Hence, we further performed the CIBERSORT algorithm to predict the immune cells infiltration between patients with IPAH and the control group. The percentage of each of the 22 types of immune cells in each sample was shown in the bar plot and heat map (Figures 7A,B). The vioplot of the immune cell infiltration difference demonstrated that IPAH patients had a higher level of CD8+ T cells, resting memory CD4+ T cells, gamma delta T cells, M1 macrophages, resting mast cells, and a lower level of naïve CD4+ T cells, monocytes, M0 macrophages, activated mast cells, and neutrophils compared with the control group (Figure 7C). A correlation heatmap of immune cells revealed that gamma delta T cells were positively related with M1 macrophages (*r* = 0.69) and CD8+ T cells (*r* = 0.67), neutrophils were positively related with monocytes (*r* = 0.62), activated NK cells were positively related with follicular helper T cells (*r* = 0.58), M1 macrophages were positively related with activated memory CD4+ T cells (*r* = 0.47), whereas resting memory CD4+ T cells were negatively related to M0 macrophages (*r* = −0.5), resting mast cells were negatively related to neutrophils (*r* = −0.49) (Figure 7D). To conclude, there is a significant difference in immune cell infiltration between patients with IPAH and controls.

### The Correlation Between *HBB, RNASE2, S100A9*, and *IL1R2* and Immune Cells

**Figure 8:**
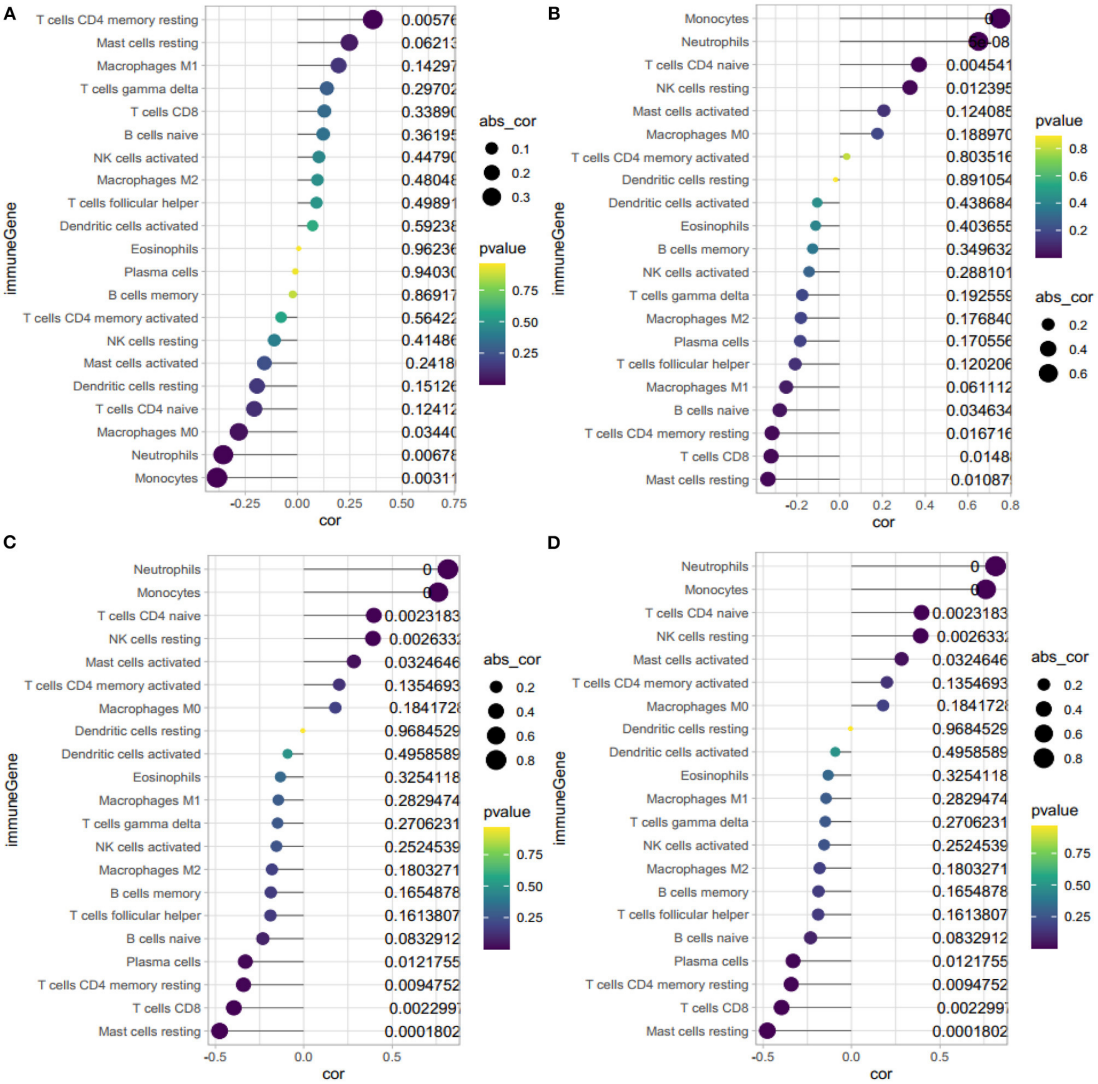
Correlation between *HBB, RNASE2, S100A9, IL1R2* and immune cells. **(A)** Correlation between *HBB* and immune cells. **(B)** Correlation between *RNASE2* and immune cells. **(C)** Correlation between *S100A9* and immune cells. **(D)** Correlation between *IL1R2* and immune cells. The size of the dots represents the strength of correlation between gene biomarkers and immune cells. The color of the dots represents the *P*-value.

The correlation analysis revealed that *HBB* (Figure 8A) had a positive correlation with memory resting CD4+ T cells (*r* = 0.36, *P* = 0.006), and negative correlation with neutrophils (*r* = −0.35, *P* = 0.007) and monocytes (*r* = −0.38, *P* = 0.003). *RNASE2* (Figure 8B) had a positive correlation with naïve CD4+ T cells (*r* = −0.37, *P* = 0.005), neutrophils (*r* = 0.65, *P* < 0.001), monocytes(*r* = 0.75, *P* < 0.001), and resting NK cells (*r* = 0.33, *P* = 0.01), and negative correlation with resting mast cells(*r* = −0.33, *P* = 0.01), CD8+T cells(*r* = −0.32, *P* = 0.01), and resting memory CD4+ T cells (*r* = −0.32, *P* = 0.02). *S100A9* (Figure 8C) had a positive correlation with monocytes (*r* = 0.76, *P* < 0.001), neutrophils (*r* = 0.82, *P* < 0.001), resting NK cells (*r* = 0.39, *P* = 0.003), naïve CD4+ T cells (*r* = 0.40, *P* = 0.002), and negative correlation with resting mast cells (*r* = −0.48, *P* < 0.001), CD8+ T cells(*r* = −0.40, *P* = 0.002), resting memory CD4+ T cells (*r* = −0.34, *P* = 0.009), and plasma cells (*r* = −0.33, *P* = 0.01). *IL1R2* (Figure 8D) had a positive correlation with monocytes (*r* = 0.43, *P* < 0.001), neutrophils (*r* = 0.66, *P* < 0.001), and negative correlation with resting mast cells (*r* = −0.32, *P* = 0.02), follicular helper T cells (*r* = −0.31, *P* = 0.02), and CD8+ T cells (*r* = −0.30, *P* = 0.02). In conclusion, *HBB, RNASE2, S100A9*, and *IL1R2* were all correlated with immune cells.

## Discussion

This study demonstrated a striking difference in the genes between IPAH and the control group. In total, 75 DEGs were identified: 35 genes were upregulated, and 40 were downregulated. We then used the LASSO regression model and SVM-RFE algorithm to select the best potential biomarkers with excellent diagnostic value: *HBB, RNASE2, S100A9*, and *IL1R2*. Moreover, the functional enrichment analysis revealed the DEGs were significantly involved in heme binding, inflammation, chemokines, cytokine activity, and abnormal glycometabolism. And we found an obvious difference in immune infiltration between patients with IPAH and controls by CIBERSORT algorithm. Furthermore, we found that these four genes were all correlated with immune cells. Previous studies have investigated the role of inflammation, chemokine, and cytokines in PAH, but as far as we known, this is the first time potential biomarkers related to immune infiltration in IPAH have been identified.

Animal models and human studies have revealed that inflammatory mediators are upregulated, including chemokines and chemokine receptors in IPAH (10, 11), such as CXCL10/CXCR3, CXCL8/CXCR1/CXCR2, CCL2/CCR2, CXCL12/CXCR4/ACKR3, CX3CL1 /CX3CR1, CCL5/CCR5/CCR1 (10, 33). The circulating cytokines were also increased in PAH, including IL-8, IL-10, IL-1β, IL-2, IL-4, IL-6, IL-12p70, IL-18, and TNFα (34). Similar to previous findings, the DEGs showed an enrichment in chemokine binding, chemokine receptor activity in this study. Other than their common role in the recruitment of immune cells in response to inflammation, chemokines are also involved in cytokine expression, pulmonary artery cell proliferation, and migration, and. Additionally, chemokines also participate in PAH progression (35, 36). And IL-10, IL-6, IL-8, and IL-12p70 have been found to predict the survival of PAH patients (34). Given the evidence that several chemokines and chemokine receptors are increased in PAH, some studies have specifically investigated pharmacological agents targeting chemokines and their receptors in different types of PAH; however, none of these agents have moved to clinical trials despite some inspiring findings.

Inflammatory cell infiltration was also observed around remodeled arteries in animal models (12) and human PAH, such as the formation of a tertiary lymphoid follicle consisting of T and B lymphocytes, and DCs have been found near remodeled arteries in IPAH, and DCs occur in the adventitia and media of muscular pulmonary arteries in human IPAH (13). A previous study observed significantly fewer CD8+ T cells and more T regulatory cells were found in the peripheral blood of patients with IPAH compared with the controls (14). In addition, mast cells were elevated in the pulmonary arteries of IPAH compared with control subjects and may participate in vascular remodeling (15). IgG antibodies from patients with IPAH expressed obvious reactivity profiles with macrovascular and microvascular endothelial cell (37) and fibroblast antigens (38). However, we showed that patients with IPAH had a higher level of CD8+ T cells, resting memory CD4+ T cells, gamma delta T cells, M1 macrophages, resting mast cells, and a lower level of naïve CD4+ T cells, monocytes, M0 macrophages, activated mast cells, and neutrophils compared with the control group. This discrepancy may be explained by the fact that CIBERSORT algorithm tends to systematically over- or under-estimate cell types despite a considerably lower estimation bias. As inflammation is involved in many aspects of IPAH, it is plausible to introduce anti-inflammatory drugs into the treatment plan for IPAH, however, studies attempted to find effective anti-inflammatory agents are lacking in IPAH (39).

Four prognostic biomarkers were established according to SVM-RFE and LASSO regression. *HBB* encodes the HBB protein, which is a key component of hemoglobin, a molecule important for oxygen delivery to tissues. Cell-free hemoglobin was increased in patients with PAH compared with healthy individuals, and positively correlated with mPAH and PVR and inversely with cardiac index in patients with PAH (40). Proteins encoded by *S100A9* and *S100A8* constitute calprotectin, a heterodimeric calcium-binding protein. They can be expressed by a broad range of cell types, such as neutrophils, monocytes, early differentiation states of macrophages, and keratinocytes. Calprotectin can induce inflammation by binding to cell surface receptors. Under normal conditions, the concentrations are very low, whereas increased obviously in inflammatory diseases (41). Calprotectin also induces apoptosis in tumors at high extracellular concentrations, whereas they facilitate proliferation of tumor cells at lower levels. Cells exposing to high concentration of S100A8/S100A9 caused caspase activation (42). In our study, *S100A9* and *S100A8* (Figure 6A) were both downregulated in IPAH compared with the control group. It is acknowledged that a cancer-like increase in cell proliferation and resistance to apoptosis is one of the characteristics of IPAH. So we speculated that the lower level of *S100A9* and *S100A8* may contribute to anti-apoptosis in IPAH, and *S100A8/S100A9* may be a potential treatment in IPAH.Rnase2 encoded by *RNASE2*, is a cytotoxic protein secreted mainly by eosinophils as well as macrophages. Rnase2 has antiviral activity and chemotactic activities *in vitro* (43, 44). Yang et al. revealed that Rnase2 can activate human DCs resulting in the production of several inflammatory cytokines, chemokines, growth factors, and soluble receptors. *RNASE2* also induced maturation of DCs (45). In this study, we found that *RNASE2* was downregulated in IPAH, and it had a positive correlation with neutrophils (*r* = 0.65) and monocytes(*r* = 0.75), we suggested that *RNASE2* in IPAH was a reflection of immune dysfunction. However, the Rnase2 level in lung tissue should be further validated by western blot. *IL1R2* is a member of the interleukin-1 receptor family, which negatively regulate the IL-1 system. The IL-1 system is associated with host responses to infections, inflammation, and activation of lymphoid cells (46, 47). *IL1R2* expression is positively regulated by several anti-inflammatory mediators, in contrast, pro-inflammatory molecules and chemokines repress *IL-1R2* expression (48). In our study, *IL1R2* was significantly downregulated in IPAH compared with the control group. We suggested that the inflammation and the high level of chemokines contributed to the reduced level of *IL1R2*. In brief, these four feature genes are associated with oxygen delivery, immune dysfunction, inflammation, and may be involved in the process of anti-apoptosis in IPAH.

However, this study has its own limitations. First, the conclusions drawn from bioinformatics analysis require RT-PCR in clinical samples to further substantiate claims. Second, there were unavoidable limitations of CIBERSORT. For example, it fails to provide *P-*values for the detection limits of individual cell types. Moreover, CIBERSORT tends to overesterestimate or underestimate some cell types systematically in spite of a considerably lower estimation bias than other methods (30).

## Conclusion

*HBB, RNASE2, S100A9*, and *IL1R2* were potential diagnostic genes in IPAH. Inflammation and immune dysfunction involves in the pathogenesis of IPAH, pharmacological agents targeting chemokines and their receptors, and anti-inflammatory drugs may be potential effective treatments in IPAH in the future.

## Data Availability Statement

Publicly available datasets were analyzed in this study. This data can be found here: https://www.ncbi.nlm.nih.gov/geo/; GSE117261, GSE48149.

## Author Contributions

HZ is the principle investigator and performed data management and bioinformatics analysis. HZ and XL conducted statistical analysis and draft the manuscript. HZ, XL, and YZ edited and revised the manuscript. All authors contributed to the article and approved the submitted version.

## Conflict of Interest

The authors declare that the research was conducted in the absence of any commercial or financial relationships that could be construed as a potential conflict of interest.
